# Molecular Evolution of Antigen-Processing Genes in Salamanders: Do They Coevolve with *MHC* Class I Genes?

**DOI:** 10.1093/gbe/evaa259

**Published:** 2021-01-27

**Authors:** Gemma Palomar, Katarzyna Dudek, Ben Wielstra, Elizabeth L Jockusch, Michal Vinkler, Jan W Arntzen, Gentile F Ficetola, Masatoshi Matsunami, Bruce Waldman, Martin Těšický, Piotr Zieliński, Wiesław Babik

**Affiliations:** 1 Institute of Environmental Sciences, Faculty of Biology, Jagiellonian University, Kraków, Poland; 2 Institute of Biology Leiden, Leiden University, The Netherlands; 3 Naturalis Biodiversity Center, Leiden, The Netherlands; 4 Ecology and Evolutionary Biology, University of Connecticut, Storrs, Connecticut, USA; 5 Department of Zoology, Faculty of Science, Charles University, Prague, Czech Republic; 6 Department of Environmental Sciences and Policy, University of Milano, Italy; 7 Laboratoire d’Ecologie Alpine (LECA), CNRS, Université Grenoble Alpes and Université Savoie Mont Blanc, Grenoble, France; 8 Department of Advanced Genomic and Laboratory Medicine, Graduate School of Medicine, University of the Ryukyus, Nishihara-cho, Japan; 9 Department of Integrative Biology, Oklahoma State University, Stillwater, Oklahoma, USA; 10 School of Biological Sciences, Seoul National University, South Korea

**Keywords:** antigen-processing genes, coevolution, MHC, molecular evolution, PSMB lineages, salamanders

## Abstract

Proteins encoded by antigen-processing genes (APGs) prepare antigens for presentation by the major histocompatibility complex class I (*MHC I*) molecules. Coevolution between APGs and *MHC I* genes has been proposed as the ancestral gnathostome condition. The hypothesis predicts a single highly expressed *MHC I* gene and tight linkage between APGs and *MHC I*. In addition, APGs should evolve under positive selection, a consequence of the adaptive evolution in *MHC I*. The presence of multiple highly expressed *MHC I* genes in some teleosts, birds, and urodeles appears incompatible with the coevolution hypothesis. Here, we use urodele amphibians to test two key expectations derived from the coevolution hypothesis: 1) the linkage between APGs and *MHC I* was studied in *Lissotriton* newts and 2) the evidence for adaptive evolution in APGs was assessed using 42 urodele species comprising 21 genera from seven families. We demonstrated that five APGs (*PSMB8*, *PSMB9*, *TAP1*, *TAP2*, and *TAPBP*) are tightly linked (<0.5 cM) to *MHC I*. Although all APGs showed some codons under episodic positive selection, we did not find a pervasive signal of positive selection expected under the coevolution hypothesis. Gene duplications, putative gene losses, and divergent allelic lineages detected in some APGs demonstrate considerable evolutionary dynamics of APGs in salamanders. Overall, our results indicate that if coevolution between APGs and *MHC I* occurred in urodeles, it would be more complex than envisaged in the original formulation of the hypothesis.

**Significance**

Coevolution between two key components of adaptive immunity, antigen-processing genes (APGs) and major histocompatibility complex class I (*MHC I*) genes, may be widespread among nonmammalian vertebrates. We used an ancient tetrapod group, salamanders, to test two expectations stemming from the coevolution hypothesis. We confirmed the tight genetic linkage between APGs and *MHC I*. However, we did not find support for pervasive adaptive evolution of APGs across the salamander phylogeny. Thus, if APGs and *MHC I* indeed coevolved in salamanders, the process would be more complex than envisaged in the original formulation of the hypothesis: Salamanders may have evolved mechanisms to reconcile coevolution with *MHC I* gene duplications, increasing the efficiency of adaptive immunity, and improving protection against pathogens.

## Introduction

The adaptive immune response is a major evolutionary innovation of vertebrates (Müller et al. 2018). Understanding its evolution has fascinated and challenged evolutionary biologists and immunologists for decades (Flajnik 2018; Flajnik and Kasahara 2010; Kaufman 2018). During the adaptive immune response in jawed vertebrates, pathogen proteins are processed into antigens that are presented on the cell surface to allow the recognition and initiation of a highly specific, targeted protective response, as well as the formation of immunological memory. Based on the antigen processing compartment, the antigen-presenting molecule, and the recognition cell, different antigen presentation pathways can be distinguished (Blum et al. 2013; Murphy and Weaver 2016). In particular, the direct presentation or endogenous pathway is triggered in most cell types by intracellular pathogens. Evidence from placental mammals suggests that antigen processing in this pathway starts in the immunoproteasome, a large proteolytic complex, in which three immunoproteasome-specific catalytic subunits PSMB8 (LMP7), PSMB9 (LMP2), and PSMB10 (MECL-1) degrade pathogen proteins to short peptides with hydrophobic carboxy-terminal residues, which are suitable ligands for major histocompatibility complex class I (MHC I) proteins (reviewed in Murata et al. 2018). These peptide antigens are translocated from the cytosol to the lumen of the endoplasmic reticulum (ER) through a channel formed by the TAP1–TAP2 (transporter associated with antigen processing 1 and 2) heterodimer (Blees et al. 2017). Within the ER, the peptide-loading complex, especially TAPBP (TAP-binding protein), positions an MHC I molecule near the TAP complex and acts as a bridge between these. This enhances TAP stability and facilitates peptide translocation. TAPBP also stabilizes empty MHC molecules and optimizes MHC loading with peptides. Once an MHC I molecule has bound a peptide, the molecule travels to the cell surface to present the antigen to cytotoxic CD8+ T cells (Murphy and Weaver 2016; Paulsson 2004).

Antigen processing proteins and MHC I interact either directly or indirectly to initiate the adaptive immune response, which sets the stage for coevolution between genes that encode them. Coevolution between antigen-processing genes (APGs) and *MHC I* gene has been thoroughly studied in chicken, inferred in the frog *Xenopus* and rat and even suggested as the ancestral gnathostome condition (Joly et al. 1998; Kaufman 1999, 2015; Ohta et al. 2006). The strength of evidence for coevolution varies among the taxa and each has peculiarities indicating that the details of the process may differ. Nonetheless, in its essence, the coevolution hypothesis posits that the properties of antigenic peptides processed by proteins encoded by APG alleles match binding properties of the *MHC I* allele co-occurring on the same haplotype. Such coevolutionary fine-tuning would increase the efficiency of antigen presentation but would also lead to the emergence of specialist (i.e., processing or presenting a restricted spectrum of antigens) APGs and *MHC I* alleles. Experimental data from chicken support this last prediction (van Hateren et al. 2013; Walker et al. 2011). In several sequenced genomes of nonmammalian vertebrates, most APGs (i.e., *PSMB8*, *PSMB9*, *TAP1*, *TAP2*, and *TAPBP*) are located very close to the *MHC I*. Tight linkage would keep particular combinations of APG and *MHC I* alleles together long enough for coevolutionary fine-tuning to occur and would also reduce the chance of generating low-fitness allele combinations (reviewed in Kaufman 2015; Ohta and Flajnik 2015). Chicken and the frog *Xenopus* have a single highly expressed *MHC I* gene and highly polymorphic APGs, with particular interlocus combinations of alleles segregating as stable haplotypes (Flajnik et al. 1999; Kaufman 2015). A single highly expressed *MHC I* gene has been proposed as a consequence of coevolution (Kaufman 2015) and constitutes an important prediction stemming from the hypothesis. Such a mode of coevolution may have profound consequences for the efficiency of adaptive immunity and fighting pathogen assault, because it would impose constraints on *MHC I* variation by selecting against gene duplication. The breakup of tight linkage between APGs and *MHC I* that occurred in mammals would have led to the emergence of monomorphic generalist APGs that provide peptides to any *MHC I* allele, which in turn might have brought about evolution of the multigene *MHC I* family and better protection against pathogens (Kaufman 2015).

APG–*MHC I* coevolution as the ancestral gnathostome condition (Kaufman 1999; Ohta and Flajnik 2015) is difficult to reconcile with the pattern found in several teleost fishes, birds, and urodele amphibians (e.g., Drews and Westerdahl 2019). For example, although at least some APGs (*TAP1*) are highly polymorphic in urodeles (Fijarczyk et al. 2016, 2018), the presence of multiple highly expressed *MHC I* genes (Fijarczyk et al. 2018) should preclude coevolution under the original formulation of the hypothesis. From 6 to 21, *MHC I* loci are expressed in the axolotl, *Ambystoma mexicanum* (Sammut et al. 1999), and from two to at least five, probably more, are highly expressed (revealed by transcriptome sequencing) and polymorphic in the *Lissotriton vulgaris* complex (Fijarczyk et al. 2018). Although it remains unclear whether all these genes represent classical *MHC I* genes, urodeles nevertheless present an intriguing pattern of *MHC I* and APG variation. High *MHC* polymorphism and the unambiguous signal of adaptive evolution in several species (Babik et al. 2009; Bos and DeWoody 2005; Fijarczyk et al. 2018) testify to the functional importance of *MHC* polymorphism in urodeles, despite previous suggestions linking a weak immune response of the axolotl with a low *MHC* polymorphism (Tournefier et al. 1998). Urodeles may have found a way to achieve duplication and expansion of *MHC I*, which could provide better protection against pathogens, while maintaining coevolution with APGs. Thus, research on this group might reveal novel mechanisms relaxing the selective constraints that coevolution imposes on adaptive immunity.

Under the coevolution hypothesis, APGs should be affected by adaptive evolution driving *MHC I* variation. Novel *MHC* variants are positively selected, which leads to their establishment in populations, while various forms of balancing selection maintain the polymorphism over extended periods (Radwan et al. 2020). Together, these processes generate a signal of adaptive evolution at phylogenetic scale detectable with standard tests for positive selection (Yang 2007). Such a signal has indeed been detected in *MHC* genes of most vertebrate species analyzed so far (Radwan et al. 2020), including urodeles (e.g., Fijarczyk et al. 2018). Similarly, even though the coevolution is an intraspecific process, adaptive evolution at functionally relevant APG codons should be detectable at phylogenetic scales if these genes coevolve with *MHC I*. Although this expectation has not been tested in nonmammalian vertebrates as molecular evolution of APGs has been studied in detail only in mammals (Forni et al. 2014), the pattern observed in some APGs is likely a result of adaptive evolution and may be related to coevolution with *MHC I*. Divergent allelic lineages have been described in several jawed vertebrates for *PSMB8*, *TAP1*, and *TAP2* (Huang et al. 2013; Kandil et al. 1996; McConnell et al. 2016; Miura et al. 2010; Namikawa et al. 1995; Nonaka et al. 2000; Ohta et al. 2003), and, in some cases, these are strongly associated with *MHC I* lineages, implying coevolution (Joly et al. 1998; Walker et al. 2011). The two lineages of *PSMB8* found in some fishes, amphibians, and reptiles could imply different specificities, thereby contributing to an expanded MHC I antigen recognition repertoire and increasing fitness of heterozygous individuals. This lineage dimorphism might be under a strong overdominance-type of balancing selection (Tsukamoto et al. 2012; but see Yamaguchi and Dijkstra 2019). In fact, *PSMB8* F type has apparently been regenerated from the *PSMB8* A type at least five times independently during tetrapod evolution (Huang et al. 2013).

In this study, we tested in the urodele amphibians two of the crucial expectations derived from the coevolution hypothesis. First, we checked whether APGs and *MHC I* are indeed tightly linked, by direct estimation of the recombination rate in a large newt pedigree. Second, we checked whether APGs show pervasive adaptive evolution across urodele phylogeny, by examining the signal of positive selection. Furthermore, we studied the possible functional role of codons under positive selection within the APG proteins, inferring their potential for interaction with other proteins (measured as surface accessibility) and their electrostatic surface charge. We also tested for the presence of divergent allelic lineages in APGs, similar to those described previously in other ectotherms.

## Materials and Methods

In addition to the APGs (i.e., *PSMB8*, *PSMB9*, *TAP1*, *TAP2*, and *TAPBP*), we analyzed several non-APGs: genes within the *MHC* region, tightly linked to *MHC I*, but not involved in antigen processing or presentation (i.e., *BRD2*, *DAXX*, *KIFC1*, *RGL2*, and *RXRBA*). The non-APGs were included as a control to check whether patterns recovered for APGs could be a simple consequence of their location within the *MHC* region.

### Linkage Analysis of APGs, Non-APGs, and *MHC* Genes

To verify linkage between APGs, *MHC*, and non-APGs and to estimate the recombination rate within the region, we used genomic DNA from a large mapping (recombinant) newt population. The mapping population consisted of lab-produced F2 generation hybrids between *Lissotriton montandoni* and *L. vulgaris*, derived from two pairs of interspecific crosses (generation P). APGs, non-APGs, *MHC I* and *II*, as well as two additional markers (*LGR4* and *RABGAP1*) were genotyped by sequencing with molecular inversion probes (MIPs, supplementary table S1, Supplementary Material online) using the procedure of Niedzicka et al. (2016). The markers *LGR4* and *RABGAP1* were mapped previously in the proximity of *MHC* (Fijarczyk et al. 2018) and were used here to orient the genes of interest along the centromere–telomere axis. Because *MHC* alleles and haplotypes segregating in the mapping population were already known (Dudek et al. 2019; Fijarczyk et al. 2018), we genotyped *MHC* using allele-specific MIPs (supplementary table S1, Supplementary Material online). Cases of ambiguous genotyping were resolved using Illumina sequencing of amplicons as described previously (Dudek et al. 2019; Fijarczyk et al. 2018). Cri-map 2.507 (Green et al. 1990) was used to estimate recombination distances between genes, order them, and identify recombinants. Details of genotyping procedure and analysis of recombination are in supplementary methods, Supplementary Material online.

### Transcriptomic Data

**Fig. 1:**
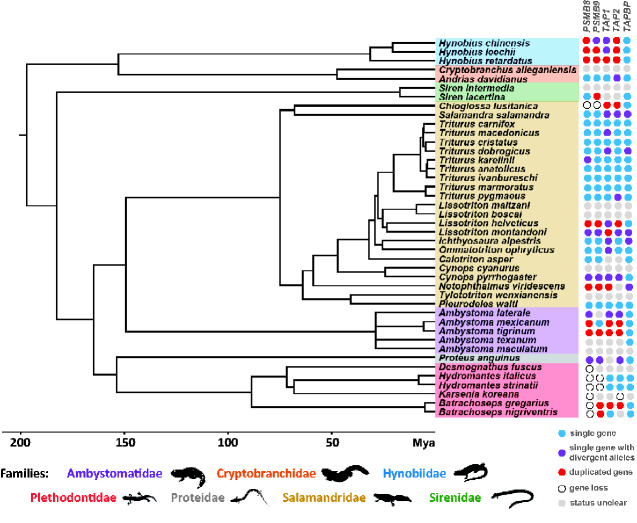
Phylogenetic tree of the species used in this study. Different colors define families. The right panel shows pattern of duplication of APGs. Blue circle indicates a single gene, whereas red circle designates a duplicated gene. Purple circle shows species with two different contigs for a gene but where three alleles were not detected in the read mapping. Empty circle indicates an apparent gene loss. Finally, gray circle indicates species in which only transcriptome assembly was available and there was no possibility to check number of alleles in the read mapping.

Transcriptome assemblies from 42 species of the Urodela representing 21 genera and seven out of nine extant families were obtained from various sources (table 1 and fig. 1). Transcriptomes of adult *Hydromantes italicus*, *Hyd. strinatii*, *Hynobius leechi*, and *Hyn. retardatus* (only larval transcriptomes have been available for this species so far, and *MHC I* expression is limited in amphibian larval stages, Salter-Cid et al. 1998) were newly generated for this study from two individuals per species, except for the *Hyd. italicus* transcriptome obtained from a single individual. RNA was extracted from tail tips stored in RNAlater; libraries were prepared with Illumina TruSeq stranded mRNA kits and sequenced on an Illumina platform, yielding 2 × 100 bp reads, except for *Hyn. leechi*, whose transcriptome was obtained using a proprietary BGI technology. Raw reads were cleaned with Trimmomatic (Bolger et al. 2014) and assembled de novo with Trinity (Grabherr et al. 2011) using settings recommended by the authors.

**Table 1:** Summary of Transcriptome Data Used in Analyses of Positive Selection

Family	Genus	Species	*PSMB8*	*PSMB9*	*TAP1*	*TAP2*	*TAPBP*	*BRD2*	*DAXX*	*KIFC1*	*RGL2*	*RXRBA*	SRA accession	References
Ambystomatidae	*Ambystoma*	*laterale*											SRR5346170	McElroy et al. (2017)
Ambystomatidae	*Ambystoma*	*maculatum*											SRR5144808	Burns et al. (2017)
Ambystomatidae	*Ambystoma*	*mexicanum*											SRR2885869	Bryant et al. (2017)
Ambystomatidae	*Ambystoma*	*texanum*											SRR5346172	McElroy et al. (2017)
Ambystomatidae	*Ambystoma*	*tigrinum*											SRR5346171	McElroy et al. (2017)
Cryptobranchidae	*Andrias*	*davidianus*											SRR5260688	Huang et al. (2017)
Cryptobranchidae	*Cryptobranchus*	*alleganiensis*											NA	Provided by D. Weisrock
Hynobiidae	*Hynobius*	*chinensis*											SRR1042328, SRR7299432	Che et al. (2014)
Hynobiidae	*Hynobius*	*leechii*											SRR13170855, SRR13170856	This study
Hynobiidae	*Hynobius*	*retardatus*											SRR13170853, SRR13170854	This study
Plethodontidae	*Batrachoseps*	*gregarius*											NA	Provided by E. Jockusch
Plethodontidae	*Batrachoseps*	*nigriventris*											NA	Provided by E. Jockusch
Plethodontidae	*Desmognathus*	*fuscus*											SRR4253224	Madison-Villar et al. (2016)
Plethodontidae	*Hydromantes*	*italicus*											SRR13170859	This study
Plethodontidae	*Hydromantes*	*strinatii*											SRR13170857, SRR13170858	This study
Plethodontidae	*Karsenia*	*koreana*											NA	Provided by T. Kwon
Proteidae	*Proteus*	*anguinus*											SRX2382497	Irisarri et al. (2017)
Salamandridae	*Calotriton*	*asper*											SRR5062019	Irisarri et al. (2017)
Salamandridae	*Chioglossa*	*lusitanica*											SRR11118089	Provided by J. W. Artzen
Salamandridae	*Cynops*	*cyanurus*											NA	Provided by D. Weisrock
Salamandridae	*Cynops*	*pyrrhogaster*											SRR2083850	Sousounis et al. (2015)
Salamandridae	*Ichthyosaura*	*alpestris*											SRX7755081	Rancilhac et al. (2021)
Salamandridae	*Lissotriton*	*maltzani*											SRR2535189, SRR2495449	Nourisson et al. (2017)
Salamandridae	*Lissotriton*	*boscai*											SRR2481123	Nourisson et al. (2017)
Salamandridae	*Lissotriton*	*helveticus*											SRR3303063, SRR7396737	Fijarczyk et al. (2016)
Salamandridae	*Lissotriton*	*montandoni*											PRJNA316531	Stuglik and Babik (2016)
Salamandridae	*Notophthalmus*	*viridescens*											SRR653294, SRR653288	Abdullayev et al. (2013)
Salamandridae	*Ommatotriton*	*ophryticus*											PRJNA498336	Wielstra et al. (2019)
Salamandridae	*Pleurodeles*	*waltl*											SRR6001111	Elewa et al. (2017)
Salamandridae	*Salamandra*	*salamandra*											SRR1693191, SRR5494542	Rodríguez et al. (2017)
Salamandridae	*Triturus*	*anatolicus*											PRJNA498336	Wielstra et al. (2019)
Salamandridae	*Triturus*	*carnifex*											PRJNA498336	Wielstra et al. (2019)
Salamandridae	*Triturus*	*cristatus*											PRJNA498336	Wielstra et al. (2019)
Salamandridae	*Triturus*	*dobrogicus*											PRJNA498336	Wielstra et al. (2019)
Salamandridae	*Triturus*	*ivanbureschi*											PRJNA498336	Wielstra et al. (2019)
Salamandridae	*Triturus*	*karelinii*											PRJNA498336	Wielstra et al. (2019)
Salamandridae	*Triturus*	*macedonicus*											PRJNA498336	Wielstra et al. (2019)
Salamandridae	*Triturus*	*marmoratus*											PRJNA498336	Wielstra et al. (2019)
Salamandridae	*Triturus*	*pygmaeus*											PRJNA498336	Wielstra et al. (2019)
Salamandridae	*Tylototriton*	*wenxianensis*											SRR2989161	Farrer et al. (2017)
Sirenidae	*Siren*	*intermedia*											NA	Provided by D. Weisrock
Sirenidae	*Siren*	*lacertina*											SRR5062014	Irisarri et al. (2017)
		*N*	33	36	38	34	40	41	41	40	36	38		

Note.—Gray cells indicate gene/species included in the analyses, total count is at the end of each column.

### Recovering Sequences of Target Genes

To identify coding sequences of our genes of interest (APGs as well as non-APGs), transcriptome assemblies of all the focal species were blasted against the reference sequences identified previously in the transcriptome of *L. montandoni*/*vulgaris* (Stuglik and Babik 2016, http://newtbase.eko.uj.edu.pl/, last accessed September 20, 2020). In rare cases, fragments of some target genes were not recovered from a transcriptome assembly. To fill these gaps, we mapped raw RNAseq reads to the reference from a closely related species and used the mapped reads to recover the sequence of the missing fragments. If substantial regions of unknown sequence remained, the gene was removed from subsequent analyses for this species. When no contig was recovered for a gene from the transcriptome assembly, the gene may have been lost in a given species, but we inferred a putative gene loss only if additional criteria were met. As genes may be missing from assemblies because of low expression level or poor quality assembly, we checked whether the gene was absent from assemblies of related species and whether its expression was comparable to that of other APGs in other species. To confirm duplications, when two transcriptome contigs were found for a gene, we visually inspected read mappings to transcripts to check whether more than two alleles were present within an individual. When more than one transcript per gene was present in the assembly, one of the sequences was picked at random for the analysis of positive selection in this gene.

Sequences of all the APGs were examined to detect the presence of divergent lineages. Phylogenetic analysis of genes with confirmed divergent lineages, *PSMB8* and *PSMB9*, was performed using all transcriptome contigs for each species and, in some cases, additional sequences recovered from read mapping, which differed by more than several bp. Maximum likelihood trees were constructed under the General Reversible Time model of evolution in MEGA7 (Kumar et al. 2016), using *Xenopus laevis* as outgroup. Node support was assessed with nonparametric bootstrapping with 1,000 replicates.

### Analysis of Positive Selection

Recombinant sequences were identified using Genetic Algorithm for Recombination Detection (GARD, Kosakovsky Pond et al. 2006). Only the break points (i.e., points that define boundaries between segments of the alignment with no evidence for ancestral recombination) supported by both the topological incongruence test (at a Bonferroni-corrected *P* value = 0.01) and the comparison of single vs. multiblock maximum likelihood models were considered. For genes with such well-supported recombination break points, analyses of selection were performed separately for each nonrecombining block.

In protein-coding sequences, nonsynonymous nucleotide substitutions change the amino acid sequence, whereas synonymous substitutions do not. The ratio (*ω*) of the rate of nonsynonymous changes per nonsynonymous site (d*N*) to the rate of synonymous changes per synonymous site (d*S*) is used to quantify the mode and strength of selection under the assumption that selection affects mainly nonsynonymous changes. When *ω* exceeds unity, positive selection that promotes changes in the protein sequence is inferred, whereas *ω* lower than unity is expected if purifying selection opposes changes in the protein sequence (Yang 2019). The overall d*N* and d*S* as well as *ω* for each of our genes of interest were calculated using MEGA7 (Kumar et al. 2016). To compare the strength of purifying selection acting on APGs versus non-APGs, we carried out a two-tailed Mann–Whitney *U* test on the values of *ω* (in genes with partitions we used weighted means for ω, taking into account the length of each partition).

To test for positive selection, the M7 codon-based substitution model that assumes a variable, beta distributed, negative selective pressure (0 ≤ *ω* ≤ 1), was compared with the M8 model that additionally assumes nonzero fraction of codons under positive selection (*ω*> 1). Both models were also compared with the null model (M0) that assumes a single *ω* for all codons. The calculations were performed in codeml from PAML 4.9 (Yang 2007). A comprehensive phylogeny of the Urodela (Jetz and Pyron 2018), modified to include the most recent phylogeny of the Salamandridae (Rancilhac et al. 2021), was used as the phylogenetic tree in all the analyses except those of *PSMB8* and *PSMB9*. For these two genes, maximum likelihood trees under the General Time Reversible model of evolution were constructed with MEGA7 (Kumar et al. 2016) based on our sequence information, because gene trees departed substantially from the species phylogeny. Models were compared with the likelihood ratio test and the Akaike information criterion. When the M8 model was supported, the Bayes empirical Bayes method (Zhang et al. 2005) was used to identify the specific codons under positive selection. In addition to codeml analyses, tests of site-level pervasive (FUBAR, Murrell et al. 2013) and episodic (MEME, Murrell et al. 2012) positive selection were performed in Datamonkey (Weaver et al. 2018).

### Protein Structure Analysis

The 3D structure of the proteins encoded by APGs in each species was modeled to locate the position of residues under positive selection and to gain insight into their possible functional significance. The structure of the protein encoded by each gene in each species was predicted using its protein sequence, the most similar protein cryo-EM structure (hereafter template) from the Protein DataBank (PDB, www.rcsb.org, last accessed June 05, 2019), and the homology modeling software Modeller (Webb and Sali 2016). For each protein sequence, we generated ten models and selected the one with the lowest Discrete Optimized Protein Energy score for further analysis. The quality of these modeled structures was checked in ModFOLD (Maghrabi and McGuffin 2017). PyMOL (Schrödinger 2019) was used to align these predicted 3D structures to the template, and its plug-in APBS to predict the electrostatic surface charge of each molecule. Because amino acid similarity between human and urodele TAPBP is <35%, we decided to be conservative and did not try to infer models for this gene based on the human template (PDB accession number 6ENY).

To shed light on the potential functional relevance of the positively selected sites, we estimated the surface receptiveness of each residue. For this, we calculated the relative solvent accessibility (RSA) from the residue’s solvent accessibility surface area (ASA) computed from the PDB models of five distantly related species: *Hyd. italicus* (Plethodontidae), *Hyn. chinensis* (Hynobiidae), *Proteus anguinus* (Proteidae), *Siren intermedia* (Sirenidae), and *Triturus dobrogicus* (Salamandridae), using the webserver xssp (http://www.cmbi.ru.nl/xssp/, last accessed August 29, 2019; based on Kabsch and Sander 1983). Then, we divided RSA by the corresponding maximum possible ASA for a given amino acid (Tien et al. 2013). We considered the residue sufficiently exposed on the protein surface to allow external interactions when the ratio was >0.2.

### *PSMB8* and *PSMB9* Population-Level Resequencing

To clarify whether the divergent *PSMB8* and *PSMB9* lineages represented divergent alleles of the same gene or duplicated genes, we designed lineage-specific MIPs (supplementary table S1, Supplementary Material online) to resequence *PSMB8* and *PSMB9* in two populations (10–21 individuals per population) of each of seven species of the genus *Triturus* (supplementary table S2, Supplementary Material online). This genus was selected because numerous samples from natural populations were available for several species (e.g., Wielstra, Burke, Butlin, and Arntzen 2017; Wielstra, Burke, Bultin, Avci, et al. 2017). The number of reads mapped to each of the two references of each *PSMB* gene (i.e., the consensus sequence of a lineage obtained from the transcriptome data of all *Triturus* species) was counted to determine the lineage/s of each individual. In the case of polymorphic populations, we used Genepop (Rousset 2008) to test whether genotype frequencies followed Hardy–Weinberg expectations, which would provide support for the presence of a single locus.

## Results

### Linkage Analysis

**Fig. 2:**
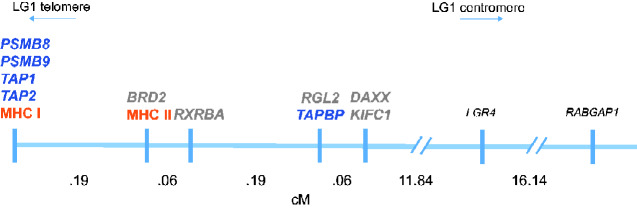
*MHC* genomic region in *Lissotriton* newts. Recombination distance is expressed in centimorgans (cM). APGs are in dark blue, non-APGs in gray, and *MHC* genes in orange. *LGR4* and *RABGAP1* are additional markers outside the *MHC* region, used to orient the genes of interest along the centromere–telomere axis.

APGs, non-APGs, and *MHC* are all tightly linked in *Lissotriton* newts. In total, eight recombinants were detected among 766 *L. montandoni* × *L. vulgaris* F2 individuals (i.e., >1,500 meioses) that formed the mapping population (supplementary table S3, Supplementary Material online). The linkage map of the *MHC* region, together with recombination distances, is given in figure 2. No recombination was detected between four APGs (*PSMB8*/*PSMB9*/*TAP1*/*TAP2*) and *MHC I*, and the map distance between this block and the most distant APG (*TAPBP*) was estimated at 0.448 cM. All five non-APGs are either embedded in this region (*BRD2*, *RXRBA*) or tightly linked to it (*DAXX*, *KIFC1*, and *RGL2*) (fig. 2). Further details are provided in supplementary results, Supplementary Material online.

### Duplication and Loss of APGs

Analyzing transcriptome assemblies, we found evidence for duplication as well as putative losses of some APGs in some species (fig. 1). In most cases, gene duplications appeared to have occurred recently, because they were not shared with closely related genera, with the possible exception of *PSMB8* and *PSMB9* (see below). The families Ambystomatidae and Hynobiidae showed frequent duplications in all APGs except *TAPBP* (fig. 1). Both *TAPBP* and non-APGs were only rarely duplicated possibly due to their location further from the remaining APGs and *MHC I* genes. Almost all cases of putative gene loss were observed in plethodontid salamanders: *PSMB8* was not found in all four genera examined, in addition, *PSMB9* was not found in *Hydromantes*, and *TAP2* was not found in *Karsenia.* The loss of *PSMB8* in plethodontids is unlikely to result from its low expression or poor transcriptome quality because *PSMB9*, a functionally related gene, was recovered at high coverage, although it has lower expression than *PSMB8* in most investigated vertebrate taxa (Petryszak et al. 2016), including the remainder of urodele species that had both genes transcribed. *PSMB8* and *PSMB9* were not recovered in *Chioglossa lusitanica*, but limited transcriptome data were available for this species, so it remains unclear whether these genes are indeed missing (table 1).

### Positive Selection and Protein Structure Analyses

Depending on the gene, from 33 to 41 species were employed in the analysis of positive selection (table 1). Only the first 2,319 bp of *DAXX* were used because the quality of alignment of the remaining part was dubious. Recombination break points were identified in *DAXX*, *KIFC1*, and *TAP2*. Alignments of these genes were divided into nonrecombining blocks, and each block was analyzed separately (table 2). *TAP2* sequences were divided into three parts with break points at alignment positions 543 and 1175. *KIFC1* and *DAXX* sequences were split into two fragments with the break point at alignment positions 1656 and 2040, respectively. The overall ratio of nonsynonymous to synonymous substitutions (*ω*) was significantly higher for APGs (*ω* = 0.20) than for non-APGs (*ω* = 0.10) (Mann–Whitney *U* = 2, *P* < 0.05). However, we did not find a consistent signal of positive selection in APGs: The M8 model, allowing positive selection, was supported only for *TAP1* and *TAP2*, whereas among non-APGs support for the M8 model was found in *BRD2* and *RGL2*. The Bayes empirical Bayes method identified two codons under positive selection in *TAP1* and *RGL2* and four in *TAP2* (table 2). FUBAR identified three codons under positive selection in *PSBM8* and *BRD2* and four in *TAP2*. More codons under episodic positive selection were localized in the MEME analysis: six in *PSMB8*, one in *PSMB9*, seven in *TAP1*, eight in *TAP2*, three in *TAPBP*, four in *BRD2*, ten in *DAXX*, four in *KIFC1*, and three in *RGL2* (table 2). However, only seven codons were identified by more than one method: three in *PSMB8* (codons 26, 104, and 190), three in *TAP2* (codons 67, 144, and 470), and one in *BRD2* (codon 8). The majority of substitutions in codons identified as positively selected were physicochemically nonconservative (supplementary table S4, Supplementary Material online), and the residues were surface accessible (supplementary table S5, Supplementary Material online), which allows them to interact with other molecules.

**Table 2.** Analysis of positive selection.

Gene	*TAP1* ^∗∗∗^			*TAP2* ^∗∗^ ^,NS,^ ^∗^			*TAPBP*			*PSMB8*			*PSMB9*			*KIFC1*			*RXRBA*			*RGL2* ^∗∗^			*BRD2* ^∗^			*DAXX*		
Length (bp)	2175			543, 633, 1026			1401			828			651			1656, 327			1359			2367			2472			2040, 279		
ΔAIC (M8-M7)	-23.82			-20.69, 2.75, -2.92			3.3			3.98			4			4, 4			0.78			-12.73			-5.14			4, 4		
dN/dS=ω	0.11/0.583=0.19			0.226/0.406=0.56 0.095/0.507=0.19 0.117/0.609=0.19			0.156/0.664=0.23			0.083/0.491=0.17			0.085/0.596=0.14			0.137/1.059=0.13 0.020/0.651=0.03			0.012/0.642=0.02			0.103/0.703=0.15			0.041/0.837=0.05			0.152/1.031=0.15 0.109/1.042=0.10		
Analysis	CODEML	FUBAR	MEME	CODEML	FUBAR	MEME	CODEML	FUBAR	MEME	CODEML	FUBAR	MEME	CODEML	FUBAR	MEME	CODEML	FUBAR	MEME	CODEML	FUBAR	MEME	CODEML	FUBAR	MEME	CODEML	FUBAR	MEME	CODEML	FUBAR	MEME
Codons		-	118^∗^			31^∗^	-	-	277^∗^	-	26^∗^	26^∗^	-	-	45^∗^	-	-	6^∗^	-	-	-		-	71^∗∗^	-	8^∗^	8^∗∗^	-	-	24^∗^
			132^∗∗^	66^∗∗^					**364** ^∗^		**104**	**104** ^∗^						37^∗^						153^∗^		65^∗^				25^∗^
			145^∗^		67	67^∗^			454^∗^			161^∗∗^						94^∗^				229^∗^					588^∗∗^			58^∗^
			292^∗^			122^∗^					190	190^∗∗^						224^∗^				233^∗^					628^∗^			106^∗∗^
			468^∗∗^	132^∗^								223^∗^												429^∗^			812^∗^			215^∗∗^
	473^∗^			144^∗^	144	144^∗^						234^∗∗^																		224^∗∗^
			664^∗^			**284** ^∗∗^																								397^∗^
			700^∗^			295^∗∗^																								416^∗^
	708^∗^					**323** ^∗^																								454^∗∗^
					**470** ^∗^	**470** ^∗^																								731^∗∗^
					476																									
				599^∗^																										

Significant support for the M8 model of positive selection (likelihood-ratio test) is indicated with asterisks following the gene symbol. For genes with the evidence of recombination(*TAP2*, *KIFC1*, *DAXX*) tests were performed for each non-recombining block separately. The ratio of nonsynonymous (dN) to synonymous (dS) substitutions was calculated in MEGA using the Nei-Gojobori method with Jukes-Cantor correction for multiple substitutions. For each gene codons identified as positively selected by three methods are indicated. Numbering of codons reflects their position in aligment. In bold codons of potential functional relevance as identified in previous research (see text for details). No asterisk: posterior probability (PP) > 0.9;

∗ p < 0.05 level, or PP > 0.95;

∗∗ p< 0.01, or PP > 0.99.

*TAP1* and *TAP2* protein structures were modeled based on the Cryo-EM structure of the human TAP ATP-Binding Cassette Transporter (PDB accession number 5U1D). This model covered residues 144–714 of the TAP1 protein alignment and 148–702 of the TAP2 protein alignment (supplementary video S1, Supplementary Material online). PSMB8 and PSMB9 protein structures were modeled based on chain K and chain N, respectively, of the Cryo-EM structure of the mouse 20S immunoproteasome (PDB accession number 3UNF). The chain K model covered residues 74–274 of the PSMB8 protein alignment and chain N model covered positions 19–217 of the PSMB9 protein alignment (supplementary video S2, Supplementary Material online). Considerable differences between urodele species in surface charge (e.g., residue 295 and 470 TAP2) and volume/shape (e.g., 470 TAP2 and 104 PSMB8) were observed at the residues under positive selection (supplementary figs. S1–S3, Supplementary Material online).

### *PSMB8* and *PSMB9* Lineages

**Fig. 3:**
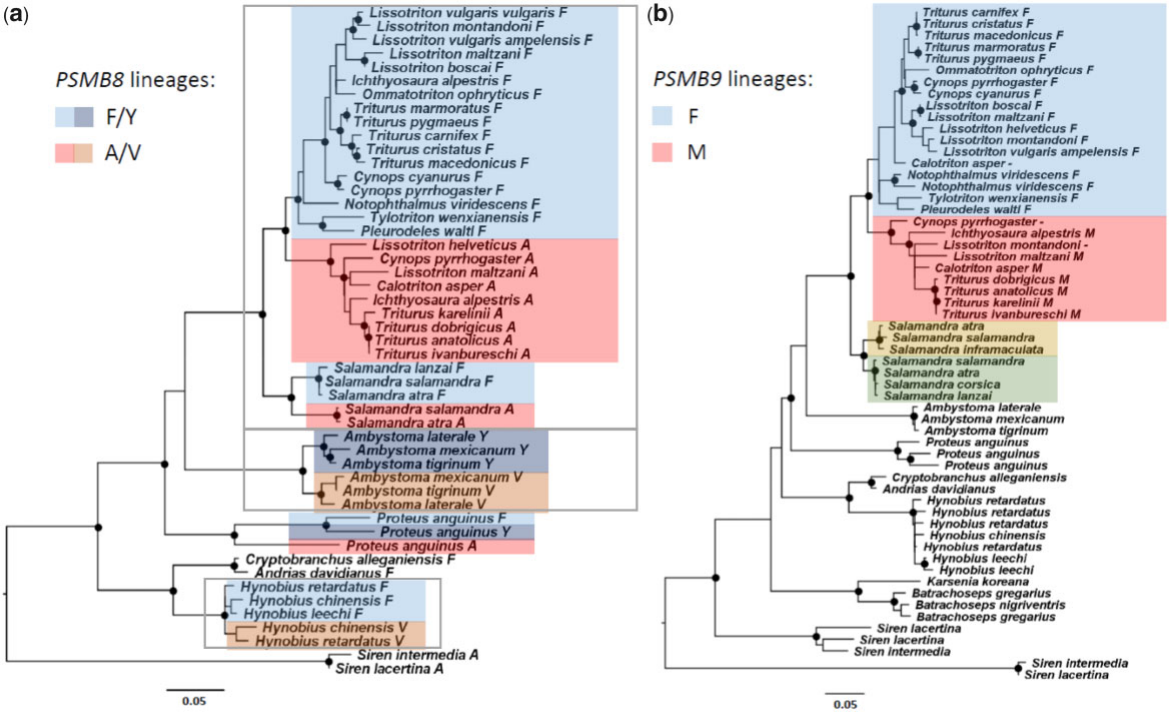
Maximum likelihood phylogenetic trees of *PSMB8* and *PSMB9*. Lineages are marked with different colors. Nodes with circles were supported by >80% bootstrap replicates. Potential lineages in the Salamandrinae *PSMB9* were marked in yellow and green. Sequences from four species of *Salamandra* from Rodríguez et al. (2017), which were not used in other analyses, were added to the trees. In *PSMB8*, gray rectangles defined the three families where relationships between species differed substantially from phylogeny, (*a*) PSMB8 lineages and (*b*) PSMB9 lineages.

At deeper levels, the maximum likelihood tree based on *PSMB8* nucleotide sequences reflected the species phylogeny; every family was recovered as monophyletic and the relationships among them were as expected. However, relationships of *PSMB8* genes within three of the families (i.e., the Hynobiidae, Ambystomatidae, and Salamandridae) differed substantially from the relationships among species (fig. 3*a*). All three of these families, and also *P. anguinus*, contained divergent lineages that correspond to the two divergent lineages (A and F) defined by codon 104 of the alignment (codon 97 in *L. montandoni* sequence and codon 31 in the tetrapod alignment of Huang et al. 2013). The amino acid that defines the clades varies between Alanine (A) and Valine (V) for the A lineage and Phenylalanine (F) and Tyrosine (Y) for the F lineage (supplementary fig. S4, Supplementary Material online). Within the Salamandridae, the deepest divergence in *PSMB8* separated the two subfamilies (i.e., the Salamandrinae and Pleurodelinae) and subfamilies were split into two clades according to the A and F lineages. Furthermore, in *P. anguinus*, the Hynobiidae, Ambystomatidae, and Salamandrinae, both the A and F lineages were detected in most species sampled. In the Pleurodelinae, this was the case at the genus level but usually only one lineage was found at species level. We detected more than two alleles, that is, gene duplication, in at least one individual of the following species: *Ambystoma mexicanum*, *A. tigrinum*, *Hyn. leechii*, *Hyn. chinensis*, *Hyn. retardatus, L. helveticus*, and *Notophthalmus viridescens* (fig. 1).

Interestingly, a similar pattern of divergent lineages appeared in the phylogenetic tree of *PSMB9* in the Salamandridae (fig. 3*b*): two clades are present in each subfamily. However, the protein sequence divergence between the clades of the Pleurodelinae is higher and widely distributed along the sequence, whereas in the Salamandrinae only two amino acids at the beginning of the protein diverged between the clades (supplementary fig. S5, Supplementary Material online). In the former subfamily, the clades could be defined by amino acid 49 of the alignment (corresponding to 48 in *L. montandoni* sequence and 31 in the alignment of Ferrington and Gregerson 2012) which was either Methionine (M) or Phenylalanine (F). This amino acid corresponds, in function and position, to the amino acid that defines the lineages in *PSMB8* (i.e., amino acid 104). These amino acids participate in defining the cleaving specificity of the immunoproteasome (Ferrington and Gregerson 2012). *PSMB9* was not found in *Hyd. italicus*, *Hyd. strinatii*, or *Chioglossa lusitanica*, although it was duplicated in at least one individual of *A. tigrinum*, *B. gregarius*, *B. nigriventris*, *Hyn. leechii*, *Hyn. retardatus*, *L. helveticus*, *N. viridescens*, and *Siren lacertina* (fig. 1).

### *PSMB8* and *PSMB9* Population Resequencing

Population-scale, genomic DNA-based resequencing revealed a single *PSMB8* and *PSMB9* lineage in five of the seven investigated *Triturus* species, in each case, it was the lineage detected earlier in the transcriptome of the species. *PSMB8* lineage F and *PSMB9* lineage F occurred in *T. cristatus*, *T. macedonicus*, *T. marmoratus*, and *T. pygmaeus*, whereas *PSMB8* lineage A and *PSMB9* lineage M were detected in *T. ivanbureschi*. Both *PSMB8* and *PSMB9* lineages were detected in *T. dobrogicus* and *T. karelinii*. In *T. dobrogicus*, individuals with both *PSMB8* and *PSMB9* lineages, as well as *PSMB8* A–*PSMB9* M individuals were present in both populations. One population, Senta (Serbia), departed from Hardy–Weinberg equilibrium with an excess of heterozygotes (individuals possessing both lineages). Together with the presence of individuals possessing three alleles, this indicates that gene duplication has occurred in *T. dobrogicus*. In *T. karelinii*, the two sampled populations exhibited different patterns. In Alushta (Crimea, Ukraine), all individuals were *PSMB8* A–*PSMB9* M, a combination revealed by transcriptome sequencing of this species, whereas in Chiantba Lake (Georgia), all individuals exhibited both lineages for both genes. This excess of heterozygotes indicated a duplication. We were not able to check whether these duplications included the whole gene or whether both loci were transcribed because transcriptomes were obtained from individuals with just one lineage per gene. Additionally, our resequencing revealed an apparently nontranscribed (judging from comparison with transcriptome data) sequence of *PSMB8*, possibly a pseudogene, covering exon 1 in *T. cristatus*, *T. macedonicus*, *T. karelinii*, and *T. dobrogicus.*

## Discussion

In this study, we tested in urodele amphibians two key predictions derived from the APGs–*MHC I* coevolution hypothesis. We found tight linkage between APGs and *MHC I*, a condition considered necessary for coevolution to operate. However, we did not find pervasive adaptive evolution in APGs across the urodele phylogeny, which would be expected under coevolution hypothesis as a consequence of adaptive evolution in *MHC I*. Nonetheless, gene duplications, gene losses, and divergent allelic lineages detected here testify to a considerable evolutionary dynamics of APGs in the Urodela, compared with other genes encoded in the same region.

### *Linkage between APGs and* MHC I

By analyzing recombination in a large experimental mapping population, we verified that APGs are tightly linked with both *MHC* classes and located closer to class I than to class II in *Lissotriton* newts. The segment containing all APGs and both *MHC* classes was shorter than 0.5 cM. Because no recombinants were observed among products of >1,500 meioses, APGs, except *TAPBP*, are exceptionally tightly linked to *MHC I*.

Adopting a high-resolution linkage approach, we estimated the recombination distances among all genes of interest, which is vital in the absence of a complete physical map of the region in any urodele. The *MHC* region remains fragmented even in the best currently available urodele genome—the chromosomal scale *Ambystoma mexicanum* assembly (Smith et al. 2019). Although the ca. 11 Mb segment of the *A. mexicanum* genome spanning from *MHC I* to *KIFC1* contains all our non-APGs, *TAPBP*, and *MHC* class II gene, it lacks most *MHC I* genes, *PSMB8*, *PSMB9*, *TAP1*, and *TAP2*, which are scattered over multiple unplaced scaffolds. From our data, we inferred that at least *PSMB8*, *TAP1*, and *TAP2* are duplicated in *A. mexicanum*, which may explain the fragmentation of the assembly. Nonetheless, the *A. mexicanum* assembly confirms the tight linkage among at least some of our genes of interest, suggesting this as an ancestral urodele condition, while showing its large physical size, exceeding 10 Mb, and apparent genomic complexity. To sum up, the tight linkage between APGs and *MHC I* confirmed in *Lissotriton*, and likely to occur also in other genera, meets a condition deemed required for coevolution between APGs and *MHC I* (Kaufman 2015).

### *Coevolution between APGs and* MHC I *in Salamanders*

Despite the tight linkage between APG and *MHC I* in *Lissotriton*, two other patterns detected in salamanders appear at odds with the coevolution hypothesis as currently formulated. First, contrary to the expectation of a single classical *MHC I* gene, multiple polymorphic, highly expressed, apparently classical *MHC I* genes are present in urodele species studied so far (Fijarczyk et al. 2018; Sammut et al. 1999). Also our rough, transcriptome-based assesement, which almost certainly underestimated the number of genes, points to multiple *MHC I* genes in most examined species (supplementary table S7, Supplementary Material online). Second, although pervasive adaptive evolution of APGs was expected, we detected only a weak signal of positive selection, restricted to only some APGs. Still, it cannot be ruled out that coevolution does occur in salamanders, but the process would have to be more complicated than previously thought. Most importantly, mechanisms allowing *MHC I* gene duplication without disrupting coevolved interactions would have to operate. Such mechanisms could be favored by selection as they would remove the constraints in flexibility imposed by having just one highly expressed *MHC I* gene without losing the high efficiency of immune response that coevolution provides. The end result would be adaptive immune response combining the benefits of multiple *MHC I* loci and coevolved combinations of APG–*MHC I* alleles (Kaufman 1999). Whether this is the case in salamanders remains an open question. A similar situation might occur in the rat (*Rattus norvegicus*), where more than one gene is expressed, at least at the mRNA level (Walter 2020), but evidence of coevolution has been demonstrated (Joly et al. 1998).

The slightly higher *ω* values in APGs compared with non-APGs suggest that the former are less constrained, leaving some space for adaptive evolution to occur, but differences between the two categories of genes are small. APGs themselves are a heterogeneous category, with strong functional links between *PSMB8* and *PSMB9* on the one hand and between *TAP1* and *TAP2* on the other hand. It is, thus, possible that the signal of positive selection detected in *TAP1* and *TAP2* stems from coevolution with *MHC I*, although the two *PSMB8* (and in some taxa *PSMB9*) lineages may still reflect coevolution, but without signal of positive selection detectable with standard tests. In addition, purifying selection appears more pervasive in *PSMB8*, *PSMB9*, and *TAPBP* than in *TAP1* and *TAP2*, at least in humans (Forni et al. 2014). If this also applies to urodeles, it might affect the detectability of their positive selection signal (Anisimova et al. 2001). Further tests of the coevolution hypothesis should examine patterns of co-occurrence of APGs and *MHC I* alleles within individuals as well as confirm the expression of several MHC I molecules at the cell surface. Finally, it remains unclear which evolutionary mechanisms are behind relatively common and predominantly recent duplications of APGs and whether they are related to *MHC I* duplications.

### Targets of Positive Selection within APGs

The residues corresponding to codons identified as positively selected might be functionally relevant, due to their interaction with other proteins or their effect on the specificity of antigen processing. The majority of these positions showed enough surface accessibility to interact with other molecules, and some exhibited variation in charge or volume that could be associated with different specificities of such interactions. In fact, two codons identified here as positively selected had previously been recognized for their crucial functional role in the protein. The amino acid at position 284 of our TAP2 alignment (human L266) has been implicated in determining substrate specificity of the protein: changes in this position can alter the epitope repertoire (Lehnert and Tampé 2017). The amino acid at position 104 of the PSMB8 alignment (position 97 in *L. montandoni* and 31 in other tetrapods, Huang et al. 2013) characterizes the two divergent PSMB8 lineages. The two lineages seem to have different specificities contributing to an expanded *MHC I* antigen recognition repertoire and increasing the fitness of heterozygous individuals (Huang et al. 2013). Another three positively selected sites may also be relevant for the functionality of the protein. The amino acid at position 364 of the TAPBP alignment is in the area forming hydrogen bonds with MHC I (human H334 and H335 Fisette et al. 2016). The codon 323 of the TAP2 alignment is within the biochemically identified substrate-binding region (human 305 within the 301–389 region, Oldham et al. 2016). Finally, the amino acid at position 470 of TAP2 is one of the residues that differentiate the two TAP2 lineages in the rat (rat N452, Ohta et al. 2003) demonstrating its importance in the functionality of the protein. The observed concordance between the signal of selection and the functional significance of these positions points to their importance in the evolutionary fine-tuning of the adaptive immune response. Interestingly, neither of the two *TAP1* codons under positive selection found previously in *Lissotriton* species (Fijarczyk et al. 2018) were confirmed in our study, similar to what was found in mammals and human populations (Forni et al. 2014). This discrepancy might reflect the different kinds of data used for the positive selection analysis and point to differences in selective pressures at different evolutionary scales: the current study used sequences from multiple divergent species, whereas Fijarczyk et al. (2018) used intraspecific polymorphism data. Therefore, there is a pressing need to complement the available data on intraspecific variation in species where coevolution has been inferred, such as chicken and *Xenopus* frogs, by studies of molecular evolution of APGs at phylogenetic scales.

### *PSMB8* and *PSMB9* Lineages

We found two divergent lineages (i.e., A and F) of *PSMB8* in the Urodela. The two highly supported clades that did not reflect the species phylogeny were present in four of the six families that possess this gene. This mirrors the pattern described by Huang et al. (2013) for several ectothermic vertebrates, in which two ancestral divergent lineages have been maintained for a long time as trans-species polymorphisms, with gene conversion leading to partial sequence homogenization between the lineages. We found a similar pattern in the phylogeny of the Salamandridae for *PSMB9—*close inspection of the sequences allowed us to characterize two divergent lineages in *PSMB9* as well. Similar evolutionary mechanisms might act on both genes because of the tight linkage between them and the interaction of their proteins in the immunoproteasome. Distinct lineages of *PSMB9* have also been described in zebrafish (McConnell et al. 2016). Population data from *Triturus* confirmed the nonrandom association of *PSMB8* and *PSMB9* lineages, *PSMB8* A with *PSMB9* M and *PSMB8* F with *PSMB9* F. This might reflect higher efficiency of the immunoproteasome catalytic subunits encoded by the respective haplotypes, in generating ligands for MHC I proteins encoded on the same haplotype, as suggested by the coevolution hypothesis (Kaufman 2015).

In some taxa, such as sharks, the divergent *PSMB8* lineages are encoded by different genes, whereas in other fish and tetrapods, including the newt *Cynops pyrrhogaster*, these *PSMB8* lineages are alleles of a single locus (Huang et al. 2013; Tsukamoto et al. 2012). However, without detailed genomic-level analysis, it is difficult to rule out their pseudoallele status (i.e., different paralogs lost from different haplotypes with the remaining genes behaving as alleles of a single locus). In our *Lissotriton* mapping population, polymorphisms in *PSMB8* and *PSMB9* segregated as alleles, but only a single lineage was present. In *Triturus* newts, a radiation of closely related species (Wielstra et al. 2019), we obtained data from transcriptome sequencing of all species and data from population-level resequencing from genomic DNA of seven species. In five of the later, we found evidence for a single lineage of both *PSMB8* and *PSMB9*, whereas in *T. dobrogicus* and *T. karelinii*, two lineages were detected. Both *T. dobrogicus* and one *T. karelinii* populations were polymorphic. The gene duplications in these species could not be confirmed in the transcriptomes and the general pattern in *Triturus* seems to be more compatible with a single locus. However, we cannot entirely rule out the possibility that the lineages represent two paralogous genes, with one of them independently lost in some *Triturus* species. Whatever the status of divergent *PSMB8* and *PSMB9* lineages, their distribution across the urodele phylogeny testifies to the existence of selective mechanisms that maintain this polymorphism, possibly in low frequency, for considerable periods of time. The process could be similar to the one described for sticklebacks, where selection sorts standing genetic variation extremely rapidly during adaptation to novel conditions (Laurentino et al. 2020). On the other hand, the relaxation of selection or its change from balancing to directional could also result in the loss of one lineage in certain species.

The apparent loss of *PSMB8* and, in some cases, also *PSMB9* in plethodontids inferred in this study might indicate the presence of an alternative way of cleaving peptides in the endogenous antigen presentation pathway of the adaptive immune response. Indeed, birds lack immunoproteasome and associated genes, including *PSMB8* and *PSMB9*, and it has been assumed that they use constitutive proteasome to cleave peptides (Kasahara and Flajnik 2019).

## Conclusions

In conclusion, our study demonstrates, for the first time in the Urodela, the tight linkage between APGs and *MHC I*, which is considered a necessary condition for their coevolution. However, we did not find the pervasive signal of adaptive evolution in APGs, expected under the coevolution hypothesis as a consequence of adaptive evolution of *MHC I*. The APGs nonetheless evolve dynamically, with frequent gene conversion and duplication in several families and gene losses in plethodontids. The lack of a widespread signal of adaptive evolution in APGs and the presence of multiple highly expressed *MHC I* genes indicate that, if coevolution between the two indeed occurs, its mechanism must be flexible enough to allow duplication of *MHC* genes, divergent lineages of *PSMB* genes or even loss of some APGs. Further insights into the presence and nature of coevolutionary processes in the urodele *MHC* might be obtained by exploring a correlation between genetic variation of APGs and *MHC I* in a comparative framework. There is also a need for studies looking at molecular evolution of APGs in taxonomic groups such as galliform birds, in which coevolution between APGs and *MHC I* was detected using intraspecific data.

## Supplementary Material

Supplementary data are available at *Genome Biology and Evolution* online.

## Supplementary Material

evaa259_Supplementary_Data
