# Erratum to: Molecular evolution of antigen processing genes in salamanders: do they coevolve with *MHC* class I genes?

**DOI:** 10.1093/gbe/evab058

**Published:** 2021-04-30

**Authors:** Gemma Palomar, Katarzyna Dudek, Ben Wielstra, Elizabeth L Jockusch, Michal Vinkler, Jan W Arntzen, Gentile F Ficetola, Masatoshi Matsunami, Bruce Waldman, Martin Těšický, Piotr Zieliński, Wiesław Babik

Genome Biol. Evol. 13(2) doi:10.1093/gbe/evaa259

In the originally published version of this manuscript, there was information missing from Table 1. This has now been corrected.

