# Supplementary material for: Molecular Evolution of Antigen-Processing Genes in Salamanders: Do They Coevolve with MHC Class I Genes?
Source: Genome Biol Evol. 2021 Jan 27;13(2):evaa259. doi: 10.1093/gbe/evaa259 (PMC7883663; doi:10.1093/gbe/evaa259)
Supplement: evaa259_Supplementary_Data [file evaa259_supplementary_data.zip › FigS4_5_PSMBVariation.pdf]

```
                                   [---------------exon1-----------------][----------exon2------------][--exon3---][-----exon4-----][--------------exon5--------------][----exon6-----]
                                                                                                       1111111111 1111111111 1111111111 1111112222 2222222222 2222222222 2222222222 2222
                                         1111 1112222222 2233333333 3344445555 5555556666 6677888999 0000011122 2344444555 6677777888 8999990001 1111222222 2333334444 5556666777 7777
                                   3456780123 5890123456 7901234567 8904890123 4567893467 8901236479 0123423917 9104568017 0101349058 9025783562 5689123467 9045781269 3482379012 3456
Lissotriton vulgaris vulgaris F    LLDLFRGPRD TLDSTEDDRG QGPCRELLGG HMALLEPARF LQSCTEHNAK IELWQHISSI STIHFPYSSF EVRKERSSAS MVIGMIQKYD DNTTPLSNSL GYYDTVEEYD GQCHTMAYYM KEIREDLHHY QSEK
Lissotriton vulgaris ampelensis F  .......... .......... ....M.M... .......... .......... .......... .......... .......... .......... ...A...... .......... .......... .....E.... ....
Lissotriton montandoni F           ...I...S.. .......... ..T....... .......... .......... .......... .......... .......... .......... .......... .......... .......... .......... ....
Lissotriton helveticus A           .......... .......... .......... ........QY .......... .......... A...A..... DMQR...... .CH...K... ...A...S.V .......... A.....T... ...SK.MYQ. ....
Lissotriton maltzani F             .......... .......... .......... ...V...... .....Q.... .......... .......... .......... .....V.... .......... .......... .......... ...SK.MYQ. ....
Lissotriton maltzani A             .......... .......... .R.S...... ........Q. .R......S. L.....V... A..RA..... .TQR...... ..H....... ...A..C..V .......... A......... ...SK.MYQ. ....
Lissotriton boscai F               .......... .......... .......... .......... .....Q.... .......... .......... .......... .....V.... .......... .......... .......... .......... ....
Triturus carnifex F                .......... .........S .......... ......A... .....H..VN .......... .......... .......... .C.......N .......... .......... .......... .......... ....
Triturus cristatus F               .......... .........S ....K..... .......... .....H.... .......... .......... .......... .C........ .......... .......... .......... .....E.... ....
Triturus dobrigicus A              ...I...... ......N..S .......... ....Q...Q. .......... ......V... A...A..... .TQR...... .CM...K... ...S..C..V .......... .......... ...SK..YQ. ....
Triturus anatolicus A              ...I...... ......N..S .......... ....Q...Q. .......... ......V... A...A..... .TQR...... .CM...K... ...S..C..V .......... .......... ...SK..YQ. ....
Triturus ivanbureschi A            ...I...... ......N..S .......... ....Q...Q. .......... ......V... A...A..... .TQR...... .CM...K... ...S..C..V .......... .......... ...SK..YQ. ....
Triturus karelinii A               ...I...... ......N..S .......... ....Q...Q. .R........ V.....V... A...A..... DTQR...... .CM...KR.. ...S..C..V .......... .......... ...SK..YQ. ....
Triturus macedonicus F             .......... ...C.....S .......... .......... .....H.... .......... .......... .......... .C........ .......... .......... .......... .....E.... ....
Triturus marmoratus F              .......... .........S .......... .......... .....Q.... .......... .......... .......... .C........ .......... .......... .......... .......... ....
Triturus pygmaeus F                .......... .........S .......... .......... .....Q.... .......... .......... .......... .C........ .......... .......... .......... .......... ....
Ichthyosaura alpestris A           ...I...... .......E.S L......... ........Q. .R..S..... ......V... A...A..... .TQR...... .CM...K... ...A..C..V .......... .......... ...SK..YQ. ....
Ichthyosaura alpestris F           .......... .........S .......... .......... .....Q.... .......... .......... .......... .C.......N .......... .......... .......... .......... ....
Calotriton asper A                 .......... ....N..... .........D ........Q. .R........ ......V... A...A..... .TQR...... .CM.K.K... ...A..C..V ........F. A......... ...SK.MYQ. ....
Ommatotriton ophryticus F          .........G .........S .......... .......... .....Q...R .......... .......... .......... .C........ .......... .......... .......... .......... ....
Notophthalmus viridis F            .......... ....N..E.S .......... ........Q. .R...D.... .......T.. .......... .......... .CM...K... ...A...... .......... .......... .......... ...N
Cynops cyanurus F                  ....L..... .......E.S .V....M... .......... .....Q.... .......... .......... .......... .C........ ...A...... .......... .......... .......... ....
Cynops pyrrhogaster F              ....L..... .......E.S .......... .......... .....Q.... .......... ...L...... .......... .C........ ...A...... .......... .......... .......... ....
Cynops pyrrhogaster A              .......... ....N.G..S .......... ..V.....Q. .R........ ......V... A...A..... .SQR...... .CM...K... ...A..C..V .......... A......... ...SKE.YQ. ....
Tylototriton wenxianensis F        ....L..... ......GEGA ...R..V... ...V...S.. .......... .......... .N.Q...... .....M.... .CV..VK... ..KS...... .......... .......... .....E.... .A..
Pleurodeles waltl F                ...VLQ.... ......G.G. ...RG..... .......... .......... .......... .......... .......... .CM..VK... ...A...... .......... .......... .....EM... ....
Salamandra salamandra F            ....L....E ..GTD..E.S .......... ........H. .......... ......V... .......... .......... .C....K... ...ASI.... .......... .........L ........Q. ....
Salamandra salamandra A            ....L....E ..GTD..E.S .......... ........H. .......... ......V... AS..AR.A.. .M..D..A.. .CMS..K... ...ASI.... S...S..... .......H.. ...SK..YR. ....
Salamandra atra F                  ....L....E ..GTD..E.S .......... ........H. .......... ......V... .......... .......... .C....K... ...ASI.... .......... .........L ........Q. ....
Salamandra atra A                  ....L....E ..GTD..E.S .......... ........H. .......... ......V... AS..A..A.. .M..D..A.. .CMS..K... ...ASI.S.. S...S..... .......H.. ...SK..YR. ....
Salamandra lanzai F                ....L....E ..GTD..E.S .......... ........H. .......... ......V... .......... .......... .C....K... ...ASI.... .......... .........L ........Q. ....
Ambystoma mexicanum V              ....LP.S.. A.GINQ.E.P L.S....... ........H. ......GS.. L.....V... ANTQV....Y .M........ .CM...K... EA.SSI.... ...NSA...E .RAY..C.VL R.VK...YQ. .A..
Ambystoma mexicanum Y              ....LS.C.. A.GINQ.E.P L.T....... Y.......H. ......G... L.....V... .DTRY....Y .M........ .C....K... EA.SSI.... ....S..D.. .RAY..C.VL R.VK...YQ. .A..
Ambystoma laterale V               ....LP.S.. A.GINQ.E.P L.S....... R.......H. ...R..GS.. L.....V... ANTQV....Y .M........ .CM...K... EA.SSI.... ...NSA..FE .RAY..C.VL R.VK...YQ. .A..
Ambystoma laterale Y               ....LP.S.. A.GINQ.E.P L.S....... ........H. ...R..GS.. L.....V... .DTQY....Y .M........ .CV..VK..N EA.SSI.... ....S..D.. .RAY.PC.VL R..K...YQ. .A..
Ambystoma tigrinum V               ....LP.S.. A.GINQ.E.P L.S....... ........H. ......GS.. L.....V... ANTQV....Y .M........ .CM...K... EA.SSI.... ...NSA...E .RAY..C.VL R.VK...YQ. .A..
Ambystoma tigrinum Y               ....LP.S.. A.GINQ.E.P L.S....... ........H. ......GS.. L.....V... .DTQY....Y .M........ .C....K... EA.SSI.... ....S..D.. .RAY..C.VL R.VK...YQ. .A..
Cryptobranchus alleganiensis F     ....ISV.K. ..GID.EETP RE..A..CE. ..V.VQ.SD. .KTF..RDV. .Q....V... ......F... ........V. .CV..VK... .A.SS..Q.. ....S..... .R....S.VL R.LK...YQ. .A..
Andrias davidianus F               ....ISV.K. ..GID.EETP RE..A..CE. ..V.VQ.SD. .KTF..RDV. .Q....V... ......F... .......... .CV..VK.F. .A.SS..Q.. ...NS..... .R....S.VL R..K...YQ. .A..
Hynobius chinensis F               ..E.ISV.Q. ..GIN.EE.P GE.WTQ.Q.. ...VVQ.SH. .KTF..RDVQ .Q....V.N. ..L....... .M........ .LV...E... .GRSS..QN. ....S..... .R....S.VL R..K...YQ. .A..
Hynobius chinensis V               ..E.ISV.K. ..GIN.EE.P GE.WAQ.Q.. ...VVQSSH. .KTFI.RDVQ .Q....V.N. A.L.V...C. .M.N...... .LM...K... .GRSS..QN. ....S..... .R..A.C.VL R..K...YQ. .AQ.
Hynobius leechi F                  ..E.ISV.Q. NQGIN.EE.P GE.WAQ.Q.. ...VVQ.SH. .KTF..RDVQ .Q....V.N. ..L....... .M........ .LV...E... .GRSS..QN. ....S..... .R....S.VL R..K...YQ. .AK.
Hynobius retardatus F              ..E.ISV.Q. ..GIN.EE.P GE.WAQ.Q.. ...VVQ.SH. .KTF..RDVQ .Q....V.N. ..L....... .M...Q.... .LV..VK... .GRSS..Q.. ....S..... .R....S.VL R..K...YQ. .A..
Hynobius retardatus V              ..E.ISV.Q. ..GIN.EE.P GE.WAQ.Q.. ...VVQSSH. .KTFI.RDVQ .Q...YV.N. A.LQV..... .M........ .LM...K... .GRSS..QN. ....S..... .R....C.VL R..K...YQ. .A..
Siren intermedia A                 ...VCG.GQ. .MG.PDGSPA LVT.HYSF.A RTP..Q.SE. VK.Y..RDL. LH.TH.V.K. D.LEA....Y .LKNK.T... .LM...E... ...SD.C..M ....S..... .RA.....V. .Q.K.EI... IQD.
Siren lacertina A                  ...VCG.GQ. .MG.PDGTPA LAT.HYSF.A RTP..Q.SE. VK.Y..RDL. LH.A..V.K. A.LEA....Y .LKNK.T... .LM...E... ...SD.C..M ....S..... .RA.....V. .Q.K.EI... IQD.
Proteus anguinus F                 ...I.P.... A.NVM.EE.L GA..GPG-TD C.......D. VK....S... ..I....T.V .DTL....CY ...N...A.. LCV..VK... N..SSI.KMM .H..S.P... .RTY..S.TL .....N.YQ. DA..
Proteus anguinus A                 .F...P.... ..TFN..E.P R...G.G-.H -.......A. ..L...S... .......... DSMFA....Y ...N...A.. FCV..VK... ...STSC..M ..P.S..... .RT.....V. .......SQF N...
Proteus anguinus Y                 M..I.P.... A.NVM.EE.P GA..GQG-AD C.......D. VK....SS.. ..I....T.V .DTMY....H ...G...A.T LCV..VK... E..SSI..NM .H..S.L... .RAY..S.VL .....N.YQ. DA..
```

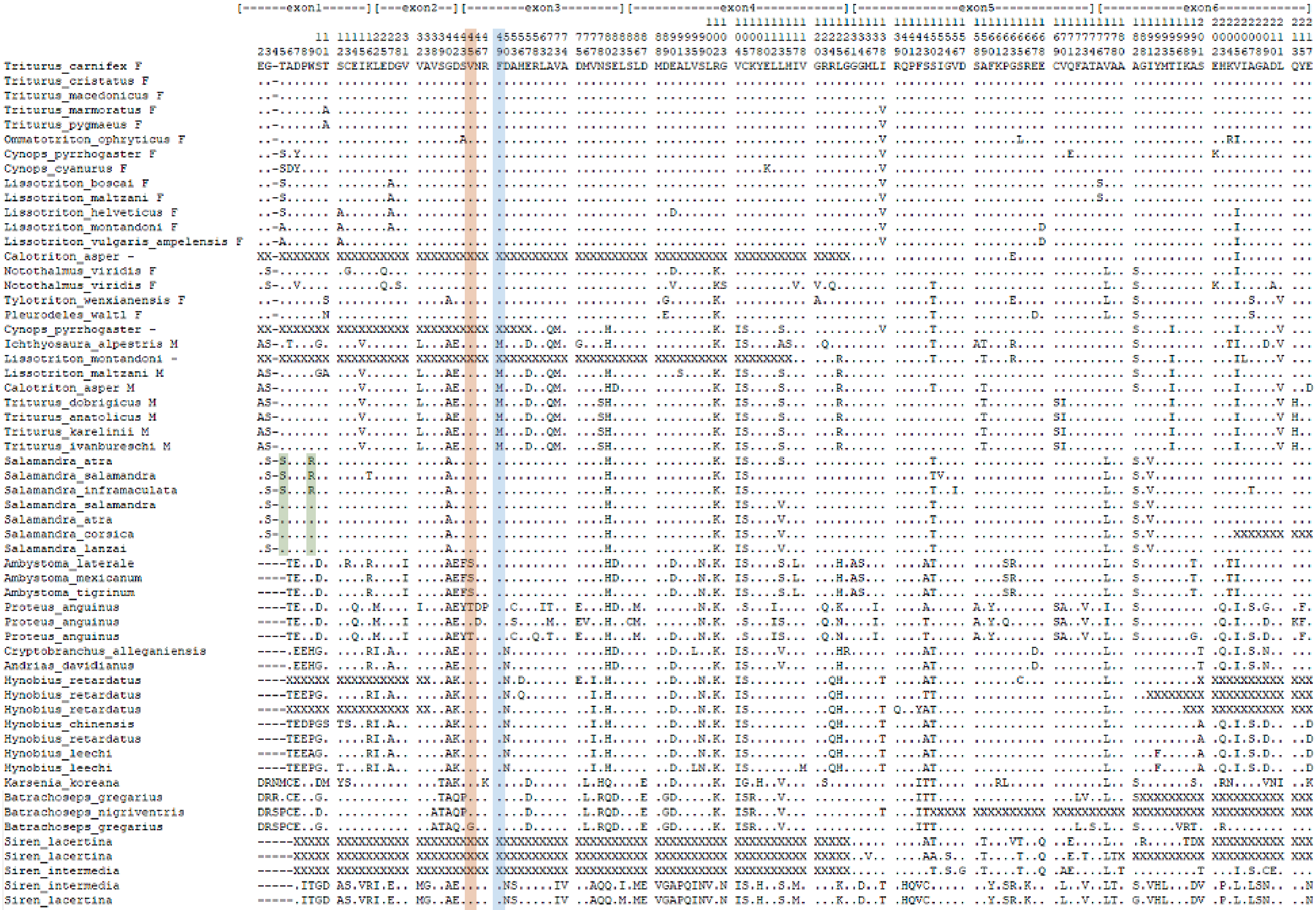

exon1
exon2
exon3
exon4
exon5
exon6
Triturus_carnifex F
Triturus_cristatus F
Triturus_macedonicus F
Triturus_marmoratus F
Triturus_pygmaeus F
Ommatotriton_ophryticus F
Cynops_pyrrhogaster F
Cynops_cyanurus F
Lissotriton_boscai F
Lissotriton_maltzani F
Lissotriton_helveticus F
Lissotriton_montandoni F
Lissotriton_vulgaris_ampelensis F
Calotriton_asper -
Notothalmus_viridis F
Notothalmus_viridis F
Tylotriton_wenxianensis F
Pleurodeles_waltl F
Cynops_pyrrhogaster -
Ichthyosaura_alpestris M
Lissotriton_montandoni -
Lissotriton_maltzani M
Calotriton_asper M
Triturus_dobrigicus M
Triturus_anatolicus M
Triturus_karelinii M
Triturus_ivanbureschi M
Salamandra_atra
Salamandra_salamandra
Salamandra_inframaculata
Salamandra_salamandra
Salamandra_atra
Salamandra_corsica
Salamandra_lanzai
Ambystoma_laterale
Ambystoma_mexicanum
Ambystoma_tigrinum
Proteus_anguinus
Proteus_anguinus
Proteus_anguinus
Cryptobranchus_alleganiensis
Andrias_davidianus
Hynobius_retardatus
Hynobius_retardatus
Hynobius_retardatus
Hynobius_chinensis
Hynobius_retardatus
Hynobius_leechi
Hynobius_leechi
Karsenia_koreana
Batrachoseps_gregarius
Batrachoseps_nigriventris
Batrachoseps_gregarius
Siren_lacertina
Siren_lacertina
Siren_intermedia
Siren_intermedia
Siren_lacertina
