# Supplementary material for: Molecular Evolution of Antigen-Processing Genes in Salamanders: Do They Coevolve with MHC Class I Genes?
Source: Genome Biol Evol. 2021 Jan 27;13(2):evaa259. doi: 10.1093/gbe/evaa259 (PMC7883663; doi:10.1093/gbe/evaa259)
Supplement: evaa259_Supplementary_Data [file evaa259_supplementary_data.zip › Supp_Meth_Result_Recomb.docx]

**Supplementary Materials to**

**Molecular evolution of antigen processing genes in salamanders: do they coevolve with *MHC* class I genes?**

G. Palomar, K. Dudek, B. Wielstra, J.W. Arntzen, E. L. Jockusch, G.F. Ficetola, M. Matsunami, B. Waldman, M. Vinkler, M. Těšický, P. Zieliński, W. Babik

**Supplementary methods**

*Linkage analysis of APG, non-APG and MHC genes*

F1 generation was obtained from two pairs of interspecific crosses, so F1 individuals were either full siblings or unrelated. Both parents (F1 generation) were known and genotyped for 235 F2 individuals, only F1 father was known and genotyped for 246 individuals and none of the parents were known for 285 F2 individuals. We used the following logic to infer partial genotypes of these unknown F1 parents. *TAPBP* had 8 distinct (multi-SNP, see below) alleles in generation P, so each founding chromosome in the pedigree was distinct. Therefore, we knew both parental *TAPBP* alleles of each genotyped F2 (there were no missing data for this gene). Thus, assuming no recombination in the region of interest in generation P, half of genotype in each gene of each F1 parent of each F2 individual was known. For each such inferred F1 individual we knew both its parents (P generation) because one founding chromosome unambiguously identified the generation P cross.

APG and non-APG reads were mapped to references using BWA-MIP [[1](#_ENREF_1)]. SNP-calling as subsequent quality filtering was performed in GATK [[2](#_ENREF_2)]. R package alleHap [[3](#_ENREF_3)] was used to identify multi-SNP alleles segregating in the mapping population, for each gene separately, using P and F1 generation individuals. Ambiguous sites identified by alleHap were excluded from allele definitions. Reconstructed alleles together with diploid sequences of all individuals were then used to reconstruct genotypes in all individuals using Phase [[4](#_ENREF_4)]. The IUPAC ambiguity codes were used in positions with probability of correct phasing below 90%. Genotypes containing ambiguity codes were compared to the sequences of known alleles and were assigned to known alleles if assignment was unambiguous, otherwise the allele was coded as missing. To estimate genotyping error 14 individuals were run in duplicates and inferred genotypes were compared between replicates. *BRD2* is duplicated in *Lissotriton*, and both gene copies have highly similar DNA sequences. Only one of these gene copies appears expressed, judging from transcriptome data (personal observation). Although our MIPs hybridized to both gene copies, we were able, using additional amplicon sequencing, to identify variants that allowed us to identify alleles of the expressed gene in MIP data and use this gene in linkage analysis.

Linkage analysis in Cri-map 2.507 [[5](#_ENREF_5)] was performed using the full pedigree, including full genotypes of known F1 parents of the F2 generation as well as inferred F1 parents. Two individuals that showed recombinant genotypes within multilocus MHC class I (see Supplementary Results) were coded as nonrecombinant on the basis of the majority haplotype to minimize the amount of missing data. In these individual recombination breakpoints were inferred between MHC class I and II, while in reality they occurred within class I.

**Supplementary Results**

*Linkage analysis*

We did not find any discrepancies between genotypes of 14 individuals analyzed in replicates. Markers differed in resolution, with some *PSMB8*, *PSMB9*, *TAP2*, *BRD2* and *RXRBA* alleles shared between generation P founders from different species (Table S6). MHC class II genotypes were unambiguous in all cases, indicating that class II haplotypes were transmitted without recombination. In class I, recombinant haplotypes were detected in two individuals (Table S3). Additionally, three individuals did not show evidence of recombination between any of the APG or non-APG genes but were either missing some alleles or had additional alleles compared to expectation of consensus haplotypes. This observation may suggest that not all class I genes are clustered in a single genomic region.

**Supplementary references**

1. Pedersen BS. 2014 Aligning sequence from molecular inversion probes. *bioRxiv*, 007260.

2. DePristo MA, Banks E, Poplin R, Garimella KV, Maguire JR, Hartl C, Philippakis AA, Del Angel G, Rivas MA, Hanna M. 2011 A framework for variation discovery and genotyping using next-generation DNA sequencing data. *Nat Genet* **43**, 491-498.

3. Medina-Rodriguez N, Santana A. 2017 alleHap: Allele Imputation and Haplotype Reconstruction from Pedigree Databases.

4. Stephens M, Smith NJ, Donnelly P. 2001 A new statistical method for haplotype reconstruction from population data. *Am J Hum Genet* **68**, 978-989.

5. Green P, Falls K, Crooks S. 1990 Documentation for CRI-MAP, version 2.4 (3/26/90). *Washington University School of Medicine, St Louis, MO*.
