# Supplementary figures and images for: Molecular Evolution of Antigen-Processing Genes in Salamanders: Do They Coevolve with MHC Class I Genes?
Source: Genome Biol Evol. 2021 Jan 27;13(2):evaa259. doi: 10.1093/gbe/evaa259 (PMC7883663; doi:10.1093/gbe/evaa259)

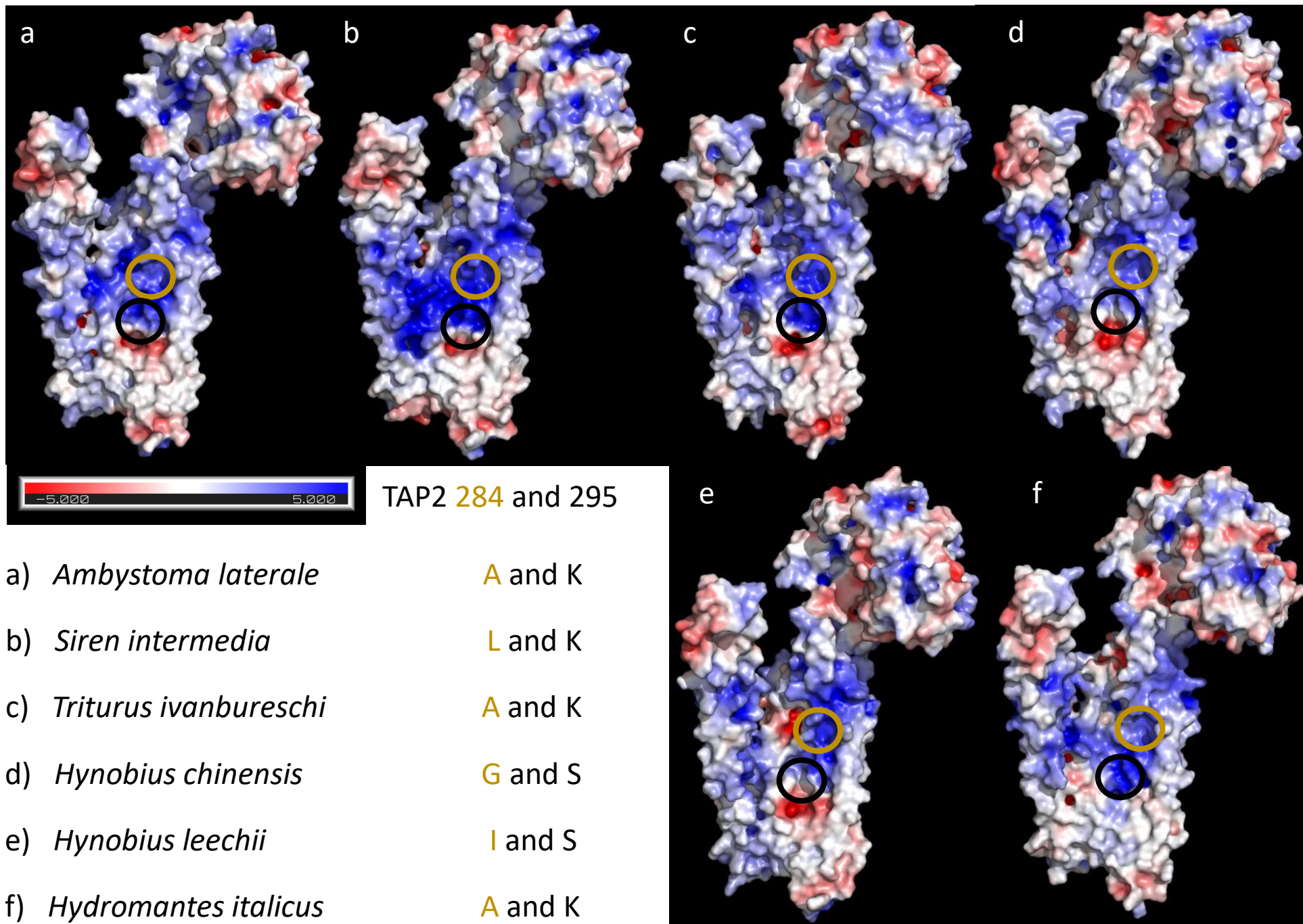

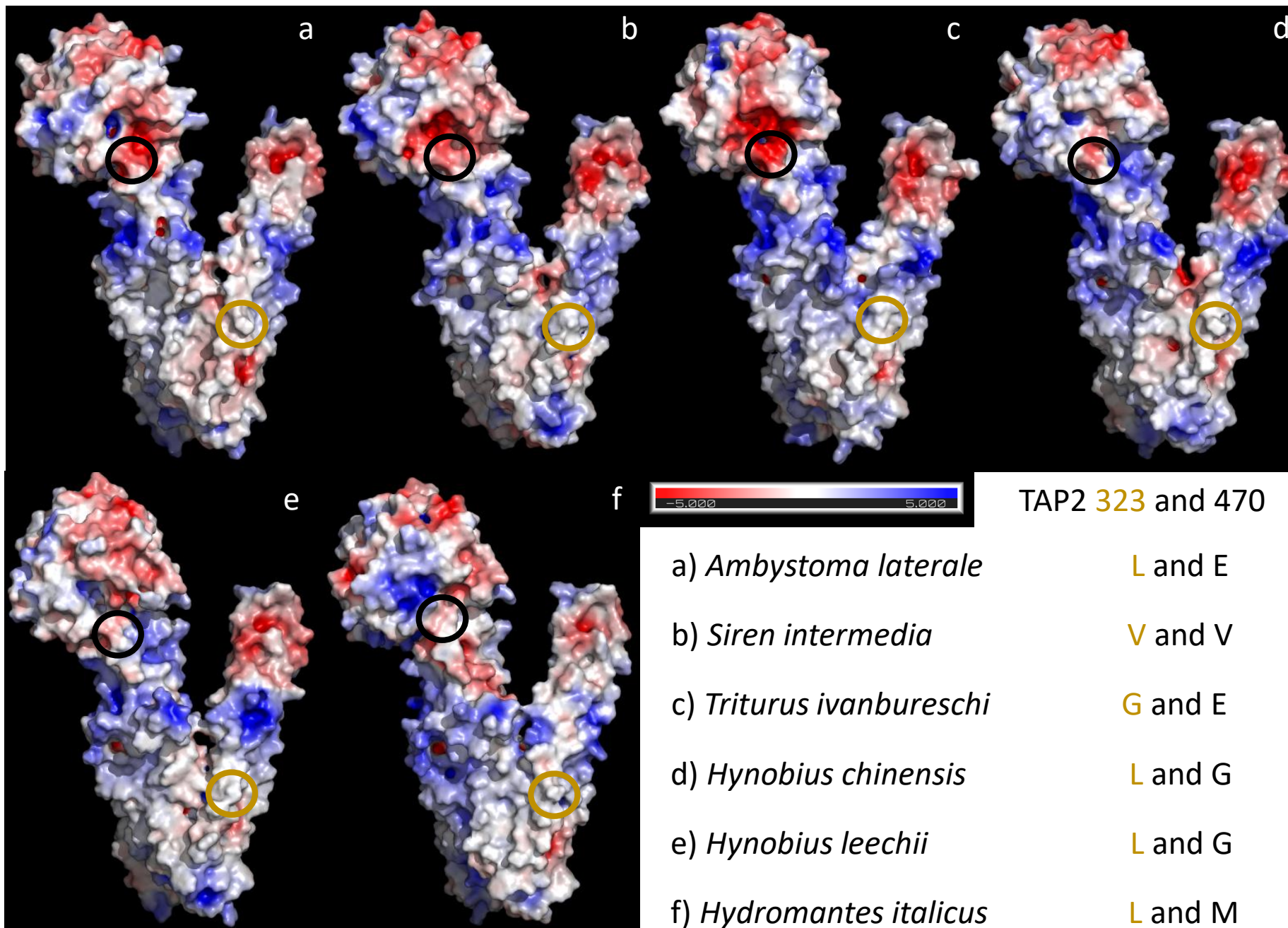

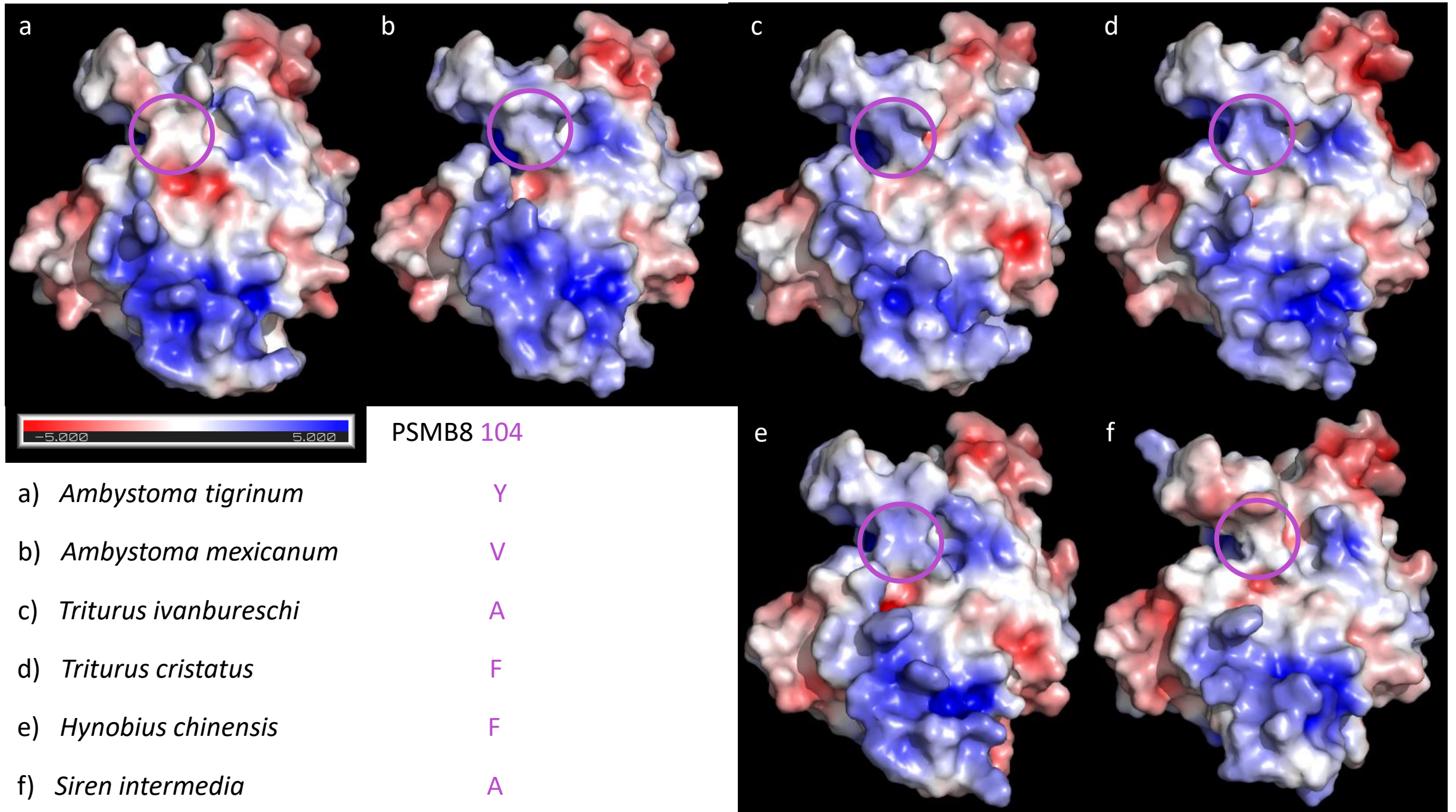

Supplement: evaa259_Supplementary_Data [file evaa259_supplementary_data.zip › Fig S1_3.pdf]
